# Supplementary material for: The shelterin component TRF2 mediates columnar stacking of human telomeric chromatin
Source: EMBO J. 2023 Dec 14;43(1):87–111. doi: 10.1038/s44318-023-00002-3 (PMC10883271; doi:10.1038/s44318-023-00002-3)
Supplement: Supplementary file 1 — Appendix [file 44318_2023_2_MOESM1_ESM.pdf]

Appendix for the paper

The shelterin component TRF2 mediates columnar stacking of human telomeric chromatin

Sook Yi Wong[1,2,†], Aghil Soman[1,†], Nikolay Korolev[1], Wahyu Surya[1], Qinming Chen[1,3], Wayne Shum[1], John van Noort[1,4], Lars Nordenskiöld[1*]

Table of content

Appendix Figure S1. DNA, protein, and nucleosome array production. S2

Appendix Figure S2. TRF2$^{\Delta N}$ binding does not evict histone octamer from Telo-10. .... S4

Appendix Table S1. Results of the MMT studies of the #Telo-18# and 601-20 arrays in the absence and the presence of TRF2$^{\Delta N}$. Fitting of the MMT data was carried out using NRL = 157 bp. S5

Appendix Table S2. Analysis of the rupture events determined in the MMT measurements of the #Telo-18# arrays in the absence and presence of TRF2$^{\Delta N}$. (See Fig 5F of the main text and Extended View Fig EV5). S5

Appendix Table S3. Fixed parameters used to fit the experimental data to the statistical mechanics model of the #Telo-18# arrays' stretching..S6

Appendix Figures

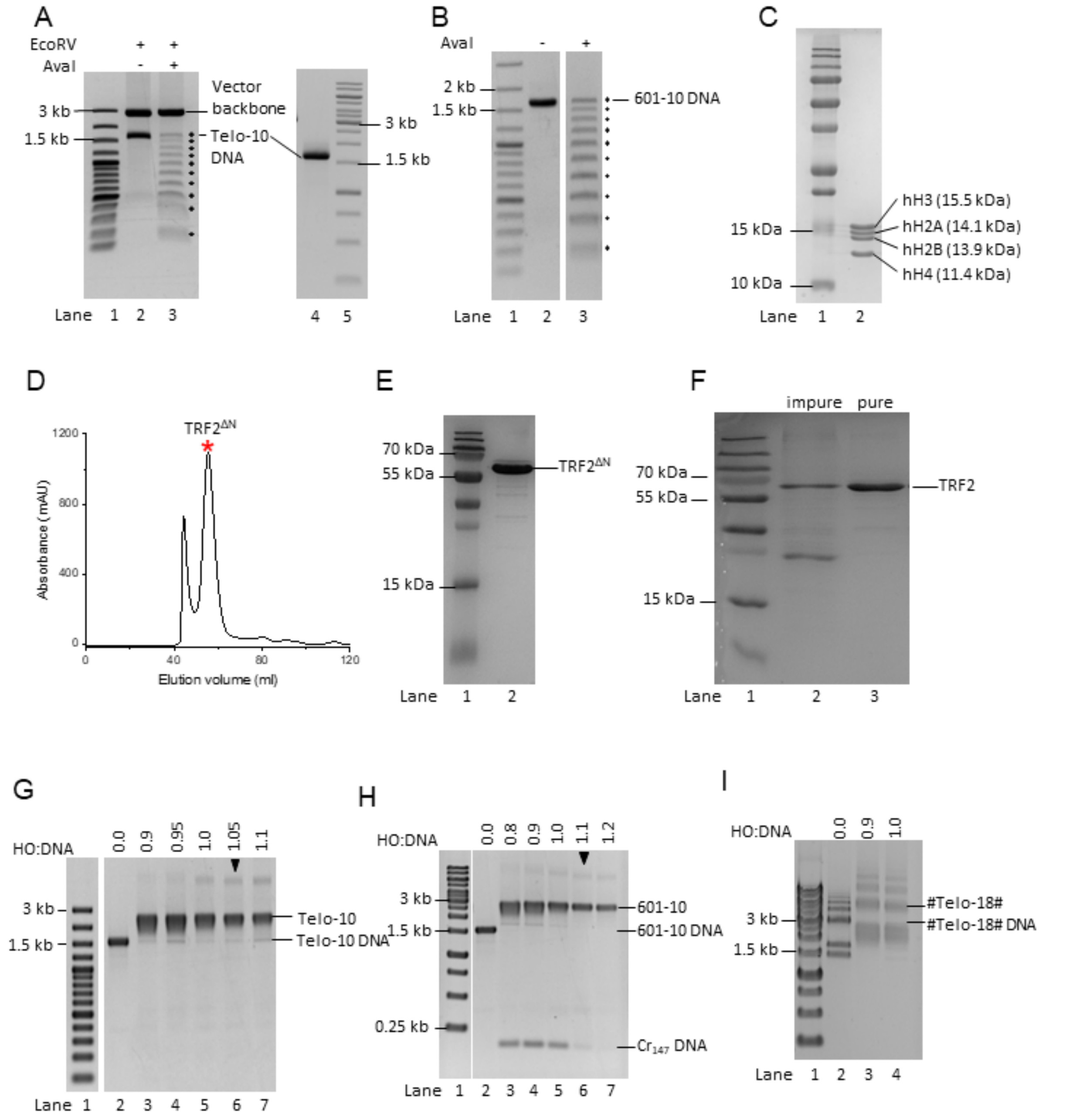

Appendix Figure S1. DNA, protein, and nucleosome array production.

A. Quality analysis of Telo-10 DNA. Left: quality analysis of Telo-10 plasmid with enzyme cleavage. Lane 1: GeneRuler 100 bp Plus DNA Ladder. Lane 2: Telo-10 plasmid cleaved with EcoRV to release the insert. Lane 3: Telo-10 plasmid cleaved with EcoRV and AvaI (partially) to reveal the number of repeats. Right: Quality of Telo-10 DNA after purification. Lane 4: Purified Telo-10 DNA. Lane 5: GeneRuler 1 Kb DNA ladder.

B. Quality analysis of 601-10 DNA. Lane 1: GeneRuler 100 bp Plus DNA Ladder. Lane 2: Purified 601-10 DNA. Lane 3: purified 601-10 DNA partially cleaved with AvaI to reveal the number of repeats.

C. Quality analysis of recombinant human histone octamer (HO) on 18% SDS PAGE. Lane 1: Precision Plus Protein™ All Blue Prestained Protein Standards. Lane 2: 0.5 µg of human HO.

D. Size-exclusion chromatogram profile of TRF2$^{\Delta N}$ purification. The second peak marked with a red asterisk is pure fractions of TRF2$^{\Delta N}$ pooled together.

E. Quality analysis of TRF2$^{\Delta N}$ verified on 18% SDS-PAGE. Lane 1: PageRuler™ Plus. Prestained Protein Ladder. Lane 2: 0.5 µg of TRF2$^{\Delta N}$.

F. Quality analysis of TRF2 verified on 15% SDS-PAGE. Lane 1: PageRuler™ Plus. Prestained Protein Ladder. Lane 2: impure TRF2. Lane 3: pure TRF2.

G. Telo-10 reconstituted with recombinant human HO titration analysed on 0.7% Tris-borate agarose gel and post-stained with SYBR® Gold Nucleic Acid Gel Stain. Lane 1: GeneRuler 100 bp Plus DNA Ladder. Lane 2: Telo-10 DNA. Lane 3-7: Titration of Telo-10 DNA with HO for the HO:DNA ratio of 0.9-1.1.

H. 601-10 reconstituted with recombinant human HO titration in the presence of competitor DNA (147 bp pUC vector backbone, Cr_{147} DNA) analysed on 0.7% Tris-borate agarose gel and post-stained with SYBR® Gold Nucleic Acid Gel Stain. Lane 1: GeneRuler 1 Kb DNA ladder. Lane 2: 601-10 DNA. Lane 3-7: Titration of 601-10 DNA with HO in the presence of Cr_{147} DNA (4% of 601-10 DNA) for the HO:DNA ratio of 0.8-1.2.

I. #Telo-18# DNA reconstituted with recombinant human HO titration analysed on 0.7% Tris-borate agarose gel and post-stained with SYBR® Gold Nucleic Acid Gel Stain. Lane 1: GeneRuler, 1 Kb DNA ladder. Lane 2: #Telo-18# DNA labelled at the DNA ends with biotin and digoxigenin. Lane 3-4: Titration of #Telo-18# DNA with HO for the HO:DNA ratio of 0.9-1.0.

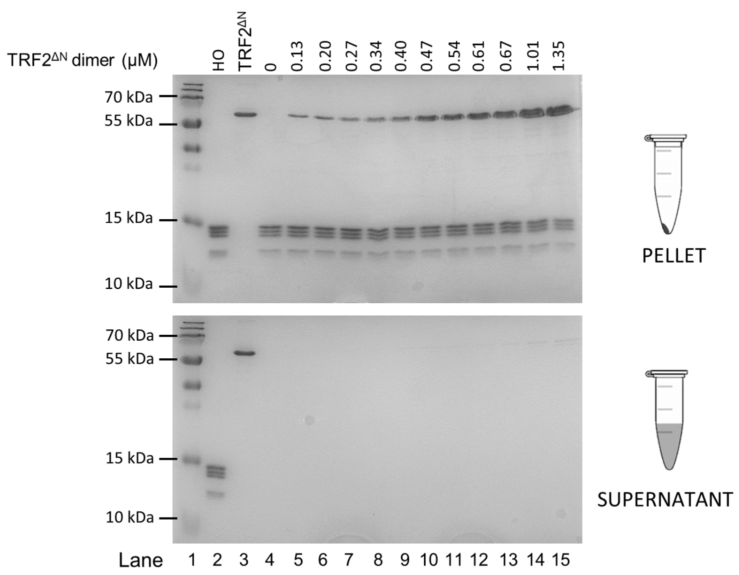

Appendix Figure S2. TRF2$^{\Delta N}$ binding does not evict histone octamer from Telo-10.

Telo-10 arrays incubated with different concentration ofTRF2$^{\Delta N}$ dimers were subjected to Mg^{2+} precipitation. The pellet (top) and supernatant (bottom) fractions were analysed on 18% SDS-PAGE. Lane 1: PageRuler™ Plus. Prestained Protein Ladder. Lane 2: histone octamer. Lane 3: TRF2$^{\Delta N}$. Lane 4-15: pellet of supernatant fraction from Mg^{2+} precipitated samples containing the indicated amount of TRF2$^{\Delta N}$ dimers.

Appendix Tables

Appendix Table S1. Results of the MMT studies of the #Telo-18# and 601-20 arrays in the absence and the presence of TRF2$^{\Delta N}$. Fitting of the MMT data was carried out using NRL = 157 bp.

TRF2$^{\Delta N}$ in the cell	Array	Nucleosomes in array	Folded nucleosomes	Stiffness, k_{fibre}, (pN/nm)	ΔG_1 (k_BT)	ΔG_2 (k_BT)	No of traces
0.0	#Telo-18#	13.5 ± 5.0	10.4 ± 3.7	0.54 ± 0.31	14.1 ± 5.4	4.5 ± 1.9	108
	601-20	17.7 ± 5.1	11.4 ± 3.9	0.55 ± 0.21	15.3 ± 5.2	4.8 ± 1.5	179
1 nM	#Telo-18#	17.0 ± 5.8	11.7 ± 4.5	0.73 ± 0.38	20.3 ± 7.1	6.1 ± 2.3	196
	601-20	17.5 ± 5.3	11.8 ± 3.7	0.55 ± 0.24	14.9 ± 4.8	4.7 ± 1.6	262
10 nM	#Telo-18#	17.6 ± 6.4	9.5 ± 3.9	0.82 ± 0.48	23.4 ± 7.9	6.2 ± 2.1	144
	601-20	18.2 ± 4.9	12.0 ± 3.7	0.54 ± 0.23	15.5 ± 5.2	4.8 ± 1.5	291
100 nM	#Telo-18#	19.7 ± 6.4	11.3 ± 4.6	0.77 ± 0.40	25.4 ± 7.9	7.3 ± 2.6	159
	601-20	18.7 ± 5.0	12.5 ± 3.9	0.64 ± 0.32	15.5 ± 5.4	4.6 ± 1.7	303

Appendix Table S2. Analysis of the rupture events determined in the MMT measurements of the #Telo-18# arrays in the absence and presence of TRF2$^{\Delta N}$. (See Fig 5F of the main text and Extended View Fig EV5).

TRF2$^{\Delta N}$ in the flow cell	Array	Slope	$F_{rupt}(0)$	d, nm	$k_{off}{\cdot}10^3$, 1/sec	Number of ruptures*
0.0	#Telo-18#	3.02 ± 0.07	10.9 ± 0.1	1.36 ± 0.03	8.9 ± 0.7	1134/624
	601-20	3.87 ± 0.05	12.8 ± 0.1	1.06 ± 0.01	9.6 ± 0.4	4817/2236
1 nM	#Telo-18#	2.64 ± 0.08	10.7 ± 0.1	1.56 ± 0.05	6.7 ± 0.7	2885/1492
	601-20	3.87 ± 0.04	12.5 ± 0.1	1.06 ± 0.01	10.1 ± 0.3	6399/3230
10 nM	#Telo-18#	2.00 ± 0.07	11.2 ± 0.1	2.05 ± 0.07	1.8 ± 0.3	2443/1122
	601-20	3.74 ± 0.04	13.1 ± 0.1	1.10 ± 0.01	8.1 ± 0.2	7724/3459
100 nM	#Telo-18#	2.77 ± 0.08	13.0 ± 0.1	1.48 ± 0.04	3.4 ± 0.4	3185/1390
	601-20	2.81 ± 0.03	11.9 ± 0.1	1.46 ± 0.02	5.2 ± 0.2	7820/3316

*First number is for all ruptures; the second is the number of ruptures used for linear fitting.

Appendix Table S3. Fixed parameters used to fit the experimental data to the statistical mechanics model of the #Telo-18# arrays' stretching.

Parameter (units)	Value
DNA contour length (bp)	3,242
Nucleosome repeat length, NRL (bp)	157
DNA persistence length (nm)	50
DNA stiffness (pN)*	1500-3500
Folded fibre length, z_0 (nm per nucleosome)	1.5
Partially wrapped length (bp per nucleosome)	55
Singly wrapped length (bp per nucleosome)	79
Free energy of single wrap rupture, ΔG_3 (k_BT)	90

*For each experimental stretching trajectory, DNA stiffness modulus was by adjusting the slope of the force-extension fitting curve to experimental data at high force (above 30-40 pN).
