# Supplementary figures and images for: The shelterin component TRF2 mediates columnar stacking of human telomeric chromatin
Source: EMBO J. 2023 Dec 14;43(1):87–111. doi: 10.1038/s44318-023-00002-3 (PMC10883271; doi:10.1038/s44318-023-00002-3)

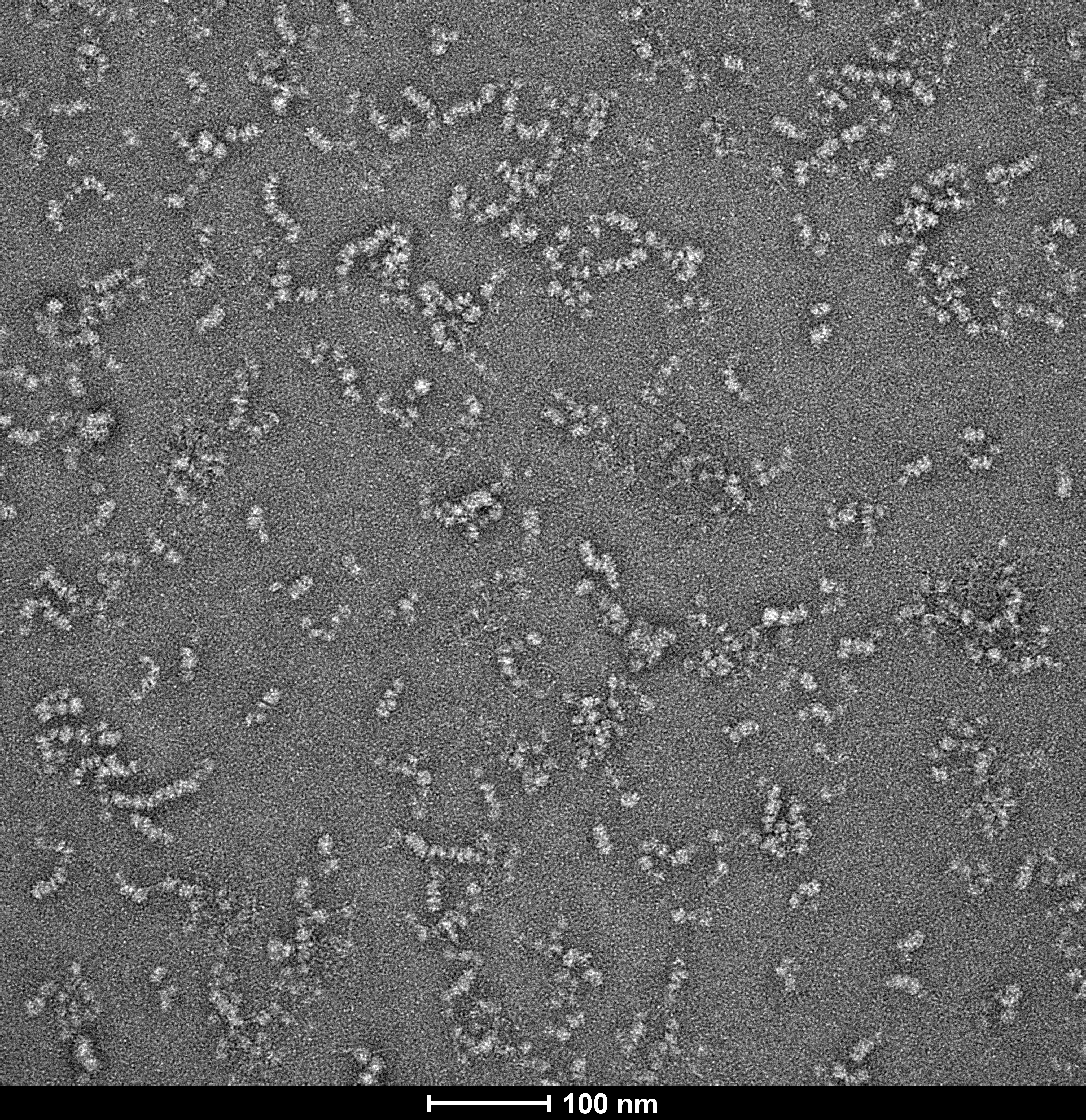

Supplement: Supplementary file 4 — Figure 2 Source Data [file 44318_2023_2_MOESM4_ESM.zip › EMBOJ-2023-114491_Source data_Figure 2/2A/Image Data_Fig 2A_Telo-10_TRF2DN.tif]

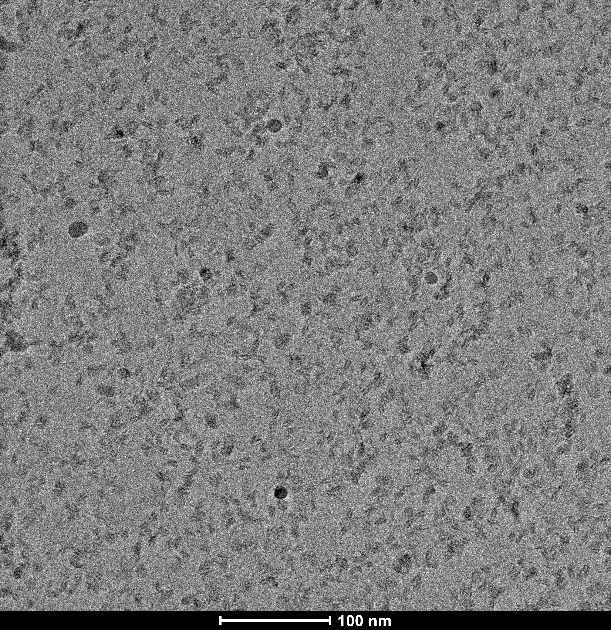

Supplement: Supplementary file 4 — Figure 2 Source Data [file 44318_2023_2_MOESM4_ESM.zip › EMBOJ-2023-114491_Source data_Figure 2/2C/Image Data_Fig 2C_Telo-4_TRF2DN dimer.bmp]

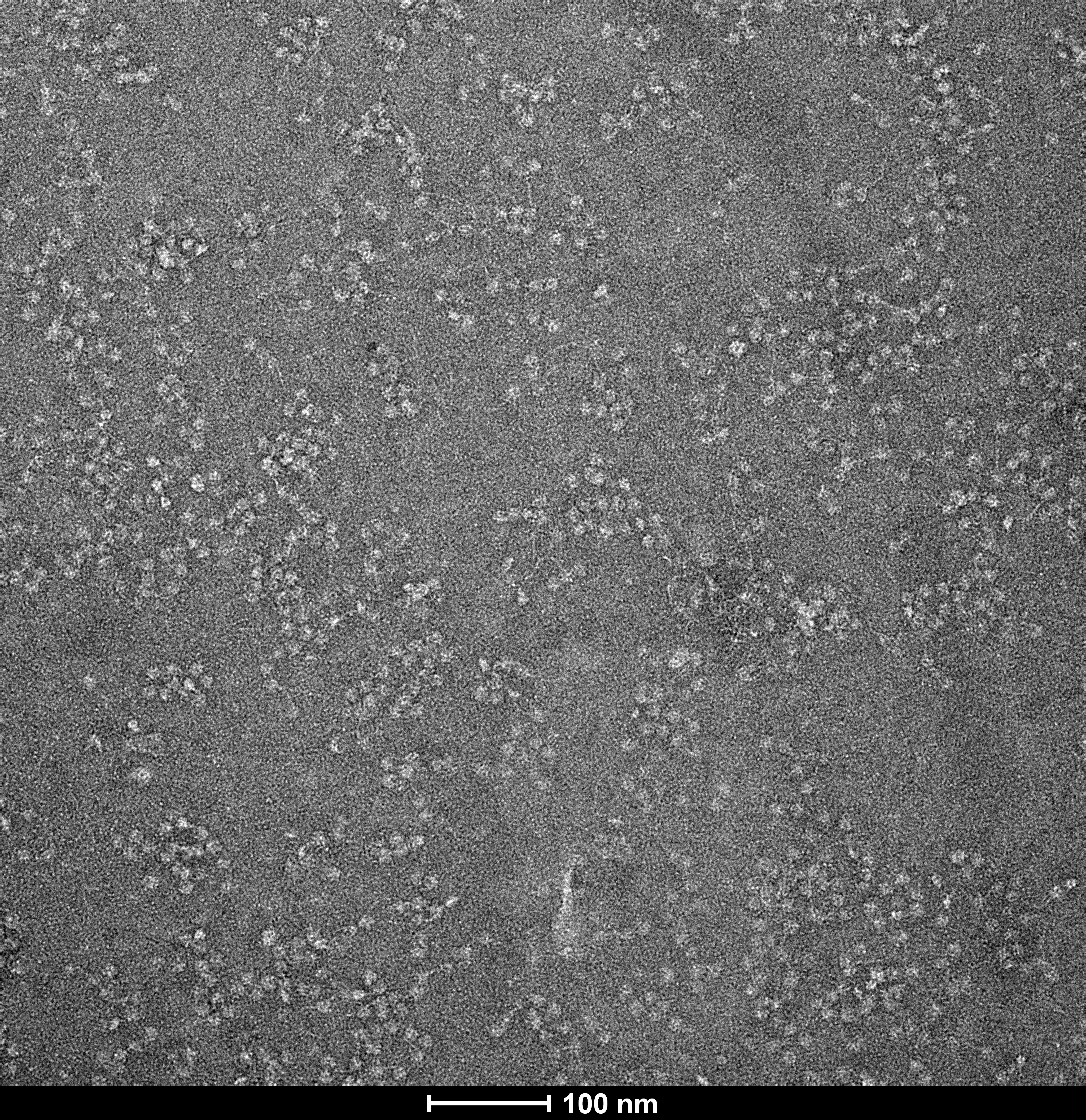

Supplement: Supplementary file 4 — Figure 2 Source Data [file 44318_2023_2_MOESM4_ESM.zip › EMBOJ-2023-114491_Source data_Figure 2/2D/Image Data_Fig 2D_601-10_TRF2DN.tif]

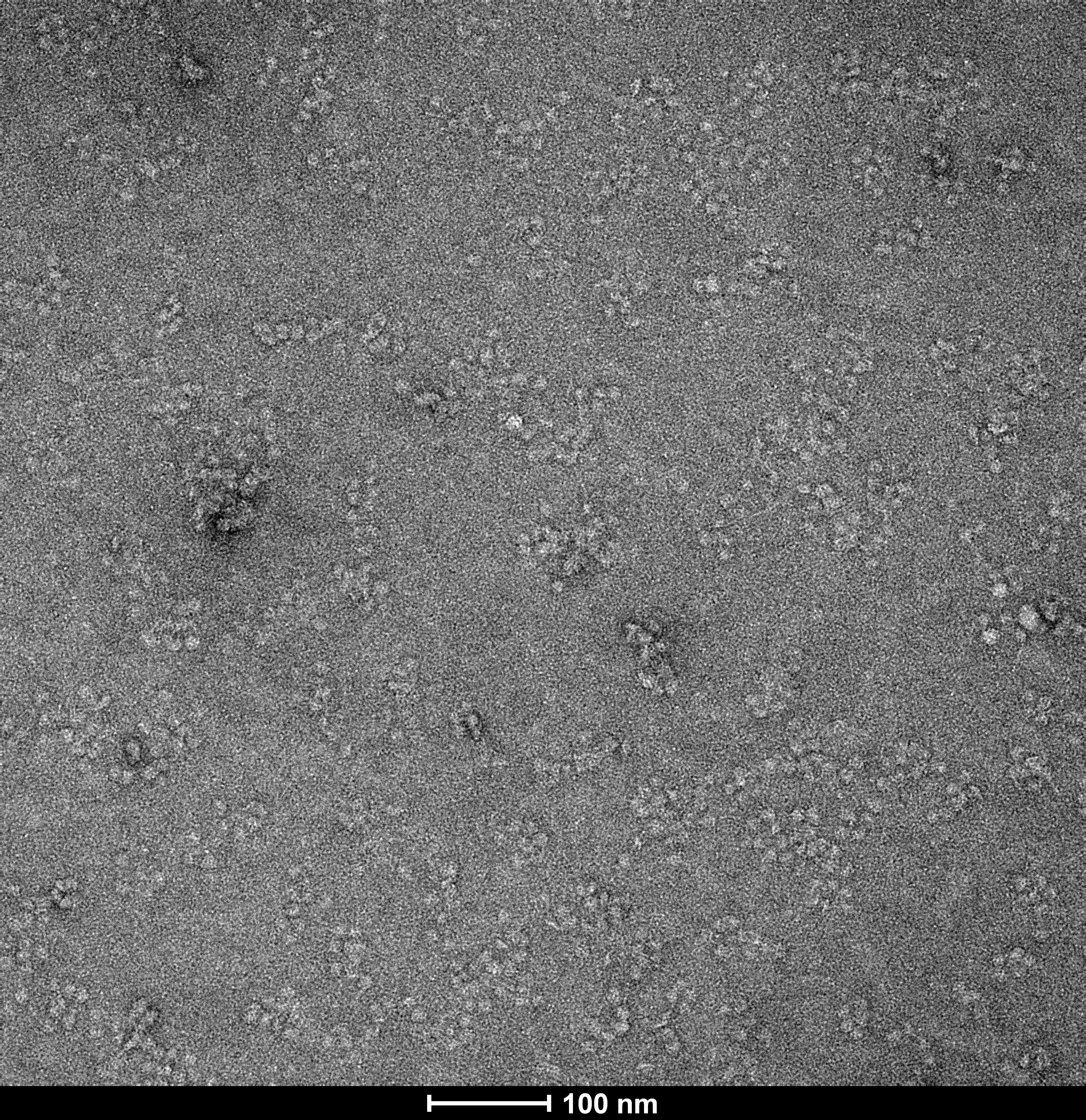

Supplement: Supplementary file 4 — Figure 2 Source Data [file 44318_2023_2_MOESM4_ESM.zip › EMBOJ-2023-114491_Source data_Figure 2/2E/Image Data_Fig 2E_601-10_Mg.tif]

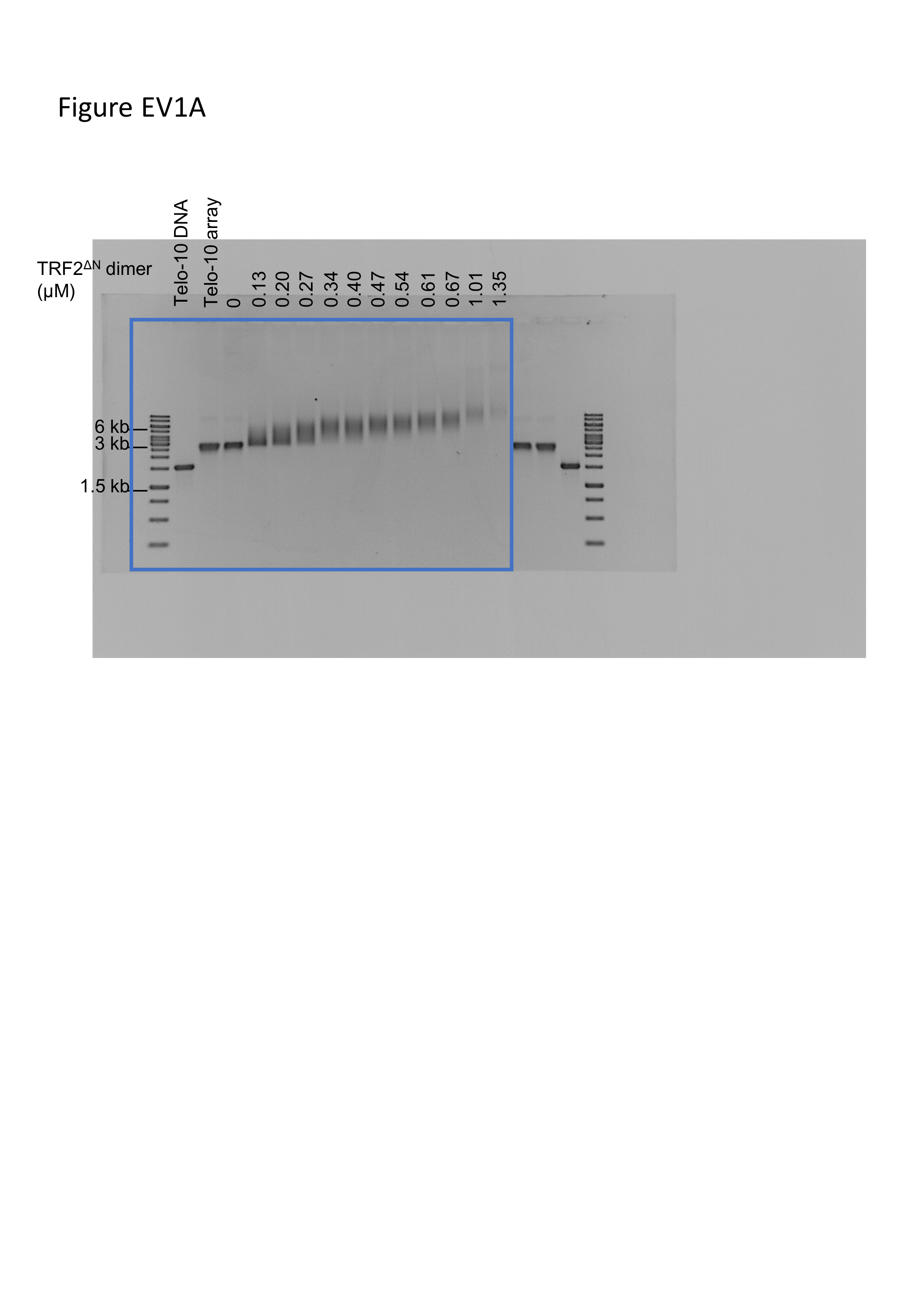

Supplement: Supplementary file 8 — Expanded View Figures Source Data [file 44318_2023_2_MOESM8_ESM.zip › EMBOJ-2023-114491_Source data_Expanded View/Figure Expanded View 1/EV1A/Image data_Figure EV1A.tif]

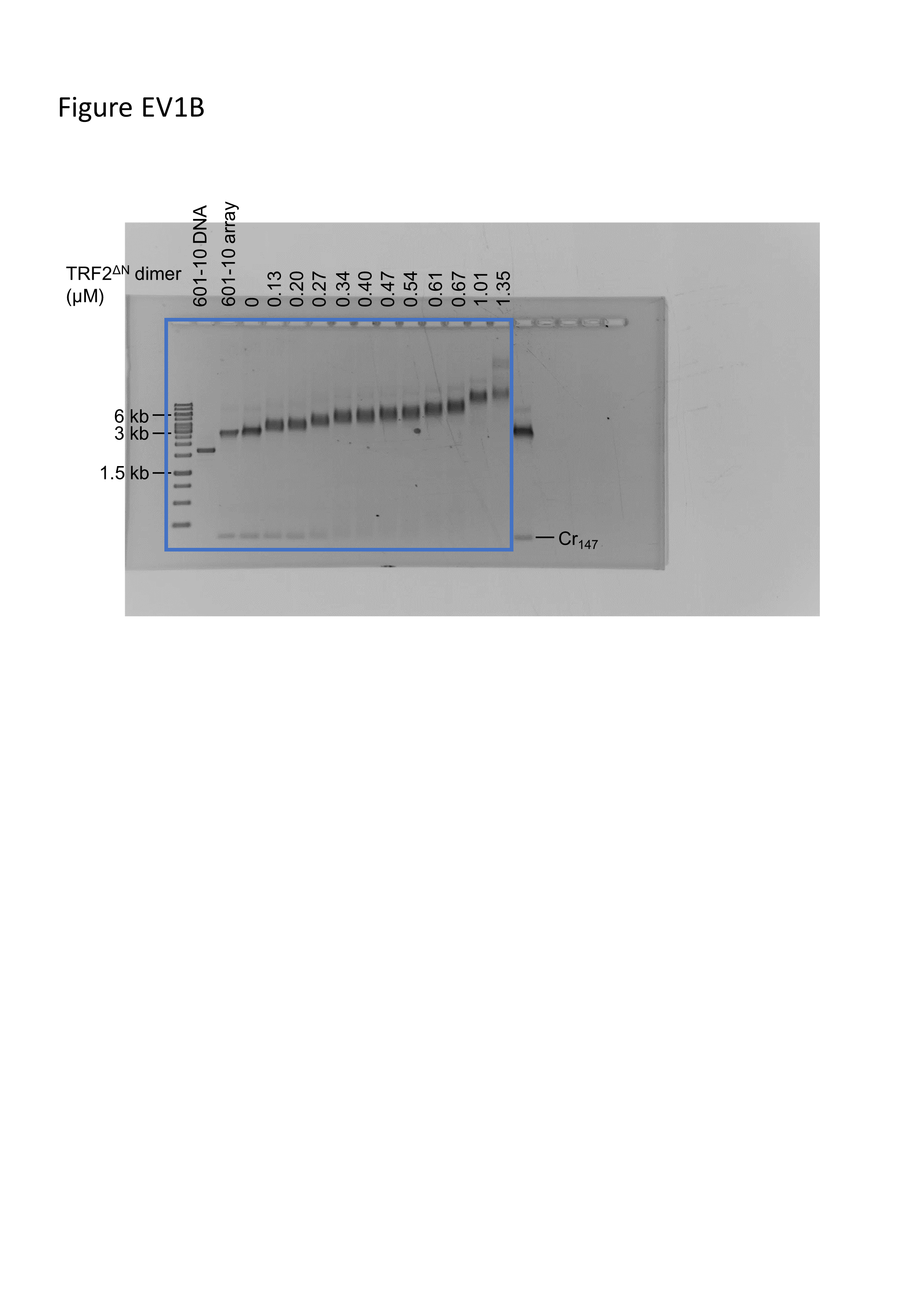

Supplement: Supplementary file 8 — Expanded View Figures Source Data [file 44318_2023_2_MOESM8_ESM.zip › EMBOJ-2023-114491_Source data_Expanded View/Figure Expanded View 1/EV1B/Image data_Figure EV1B.tif]

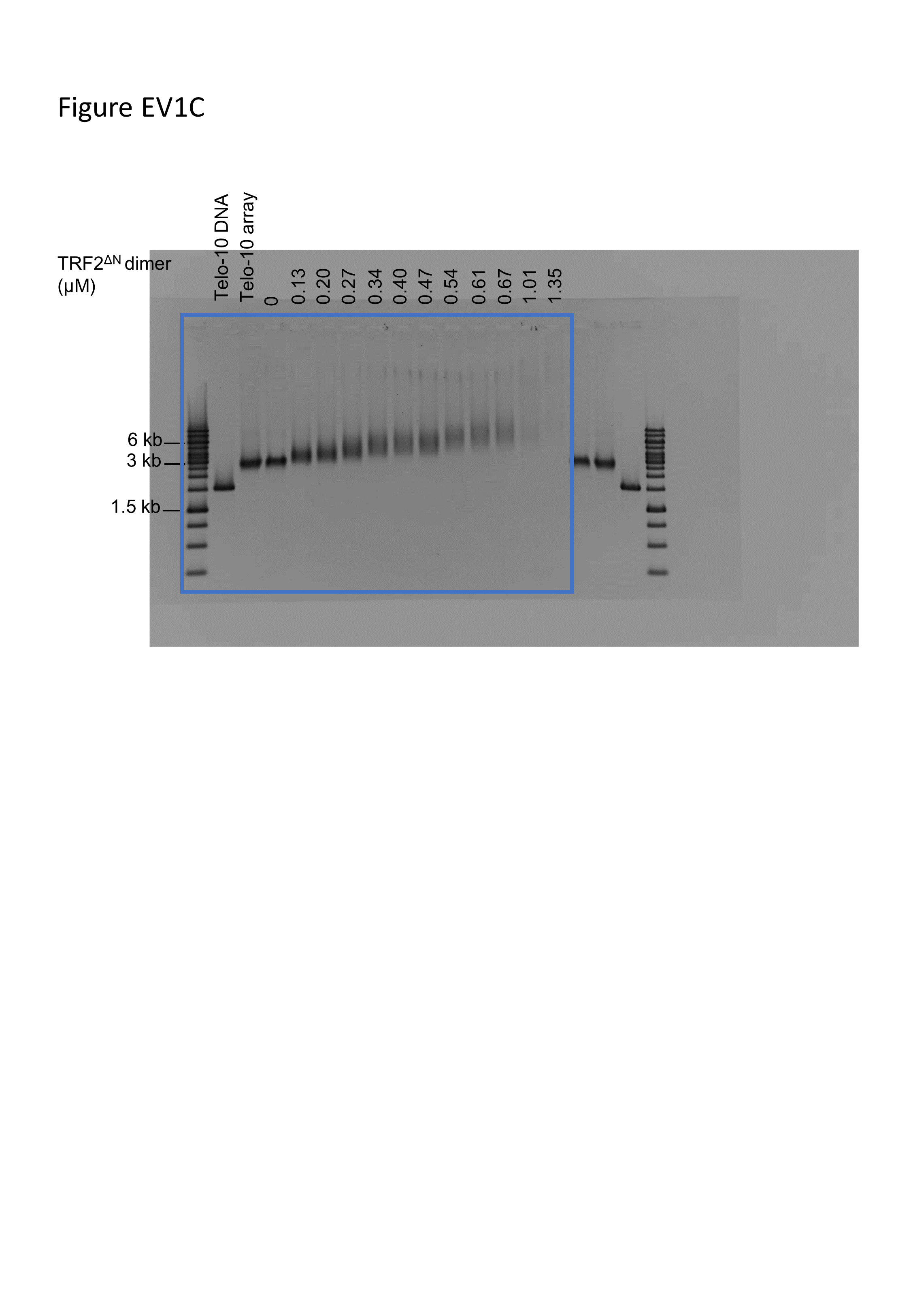

Supplement: Supplementary file 8 — Expanded View Figures Source Data [file 44318_2023_2_MOESM8_ESM.zip › EMBOJ-2023-114491_Source data_Expanded View/Figure Expanded View 1/EV1C/Image data_Figure EV1C.tif]

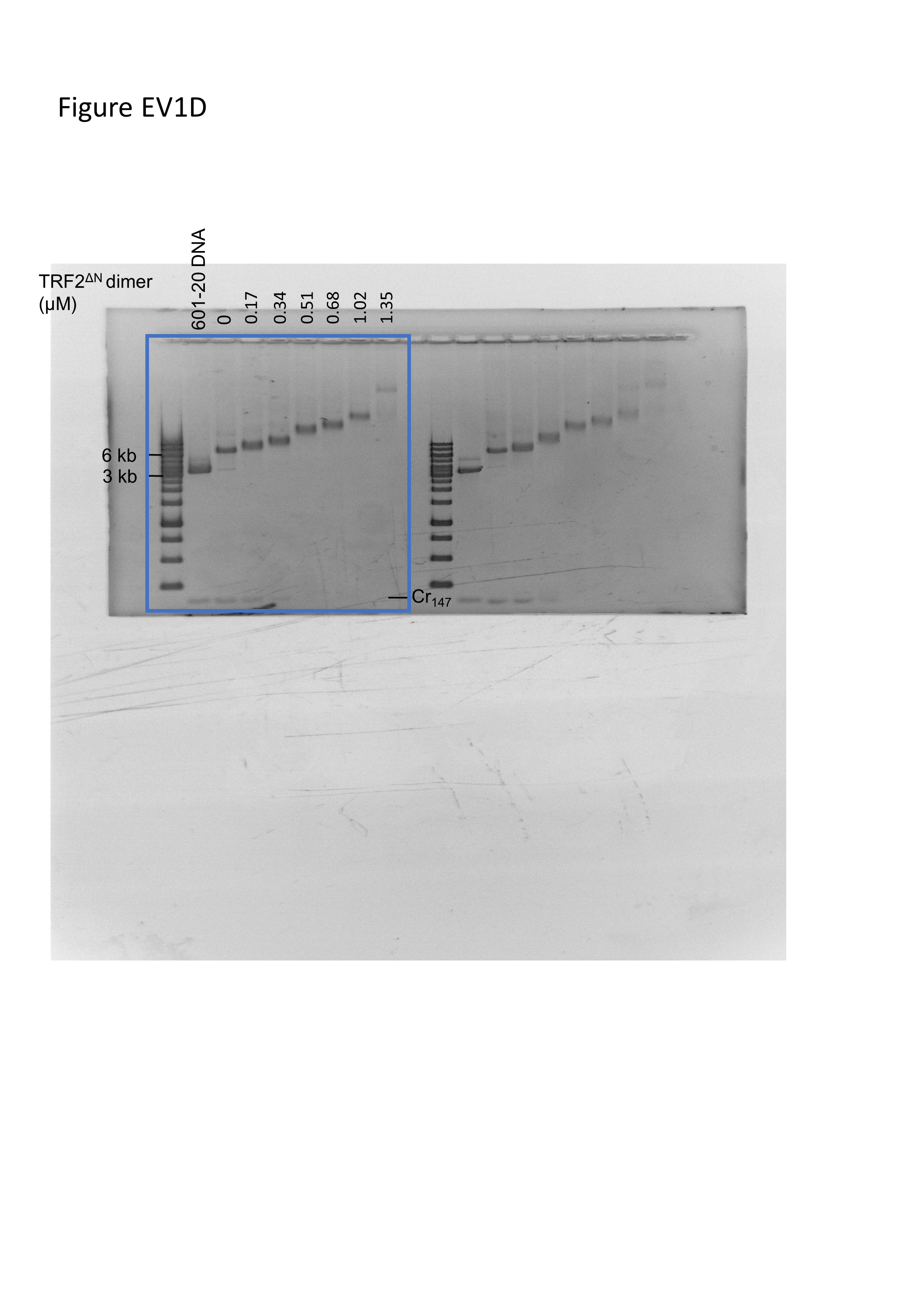

Supplement: Supplementary file 8 — Expanded View Figures Source Data [file 44318_2023_2_MOESM8_ESM.zip › EMBOJ-2023-114491_Source data_Expanded View/Figure Expanded View 1/EV1D/Image data_Figure EV1D.tif]

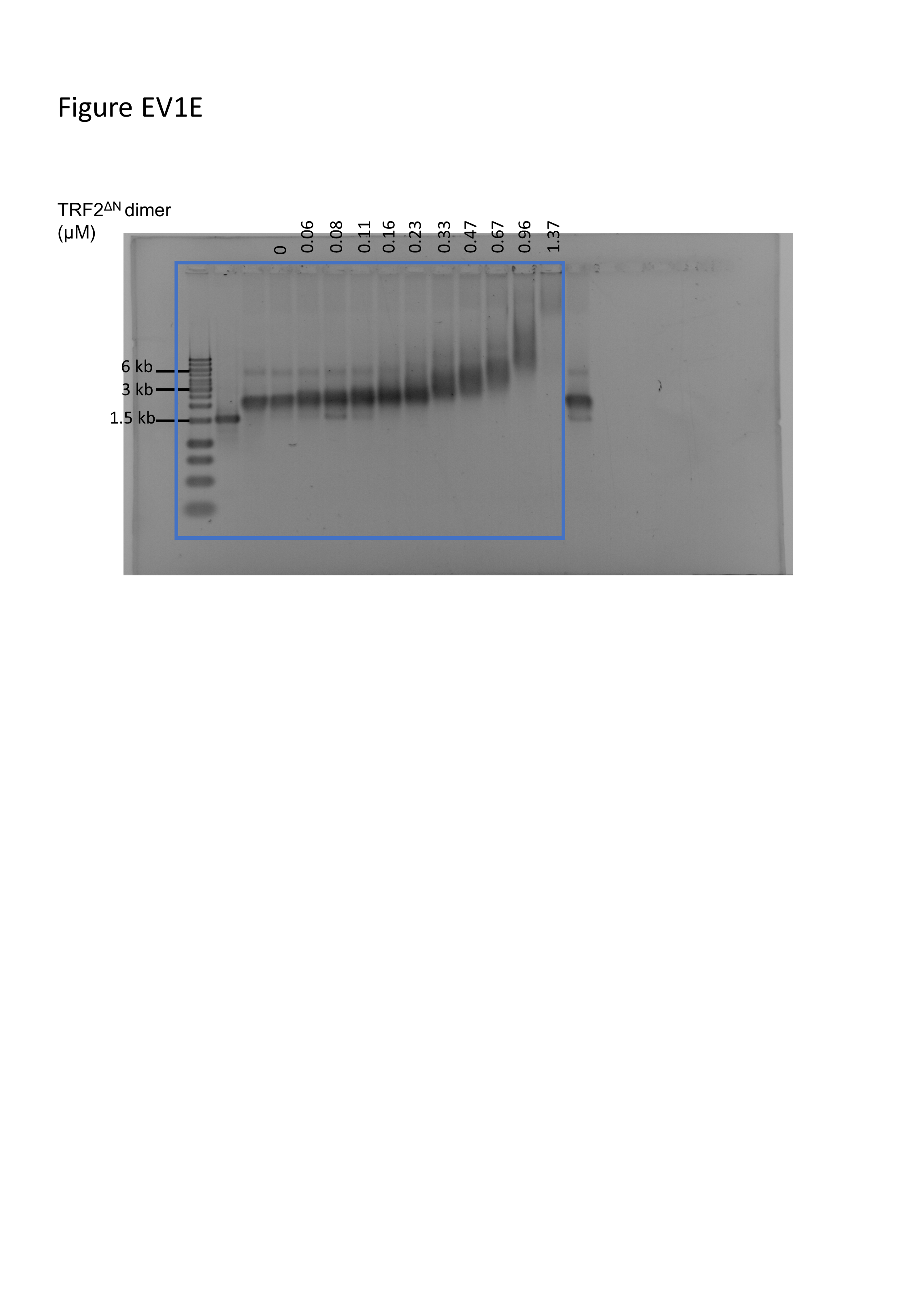

Supplement: Supplementary file 8 — Expanded View Figures Source Data [file 44318_2023_2_MOESM8_ESM.zip › EMBOJ-2023-114491_Source data_Expanded View/Figure Expanded View 1/EV1E/Image data_Figure EV1E.tif]

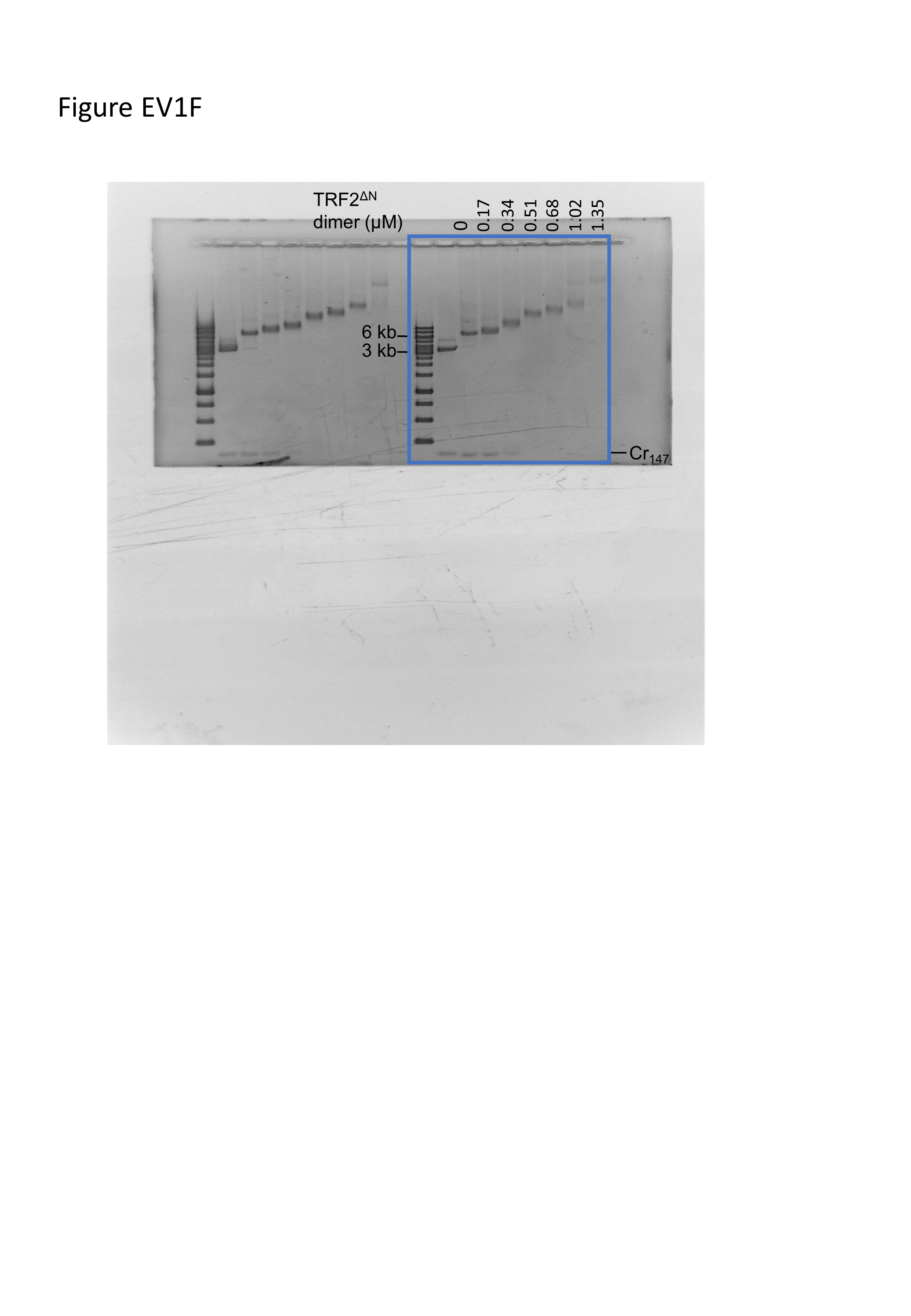

Supplement: Supplementary file 8 — Expanded View Figures Source Data [file 44318_2023_2_MOESM8_ESM.zip › EMBOJ-2023-114491_Source data_Expanded View/Figure Expanded View 1/EV1F/Image data_Figure EV1F.tif]

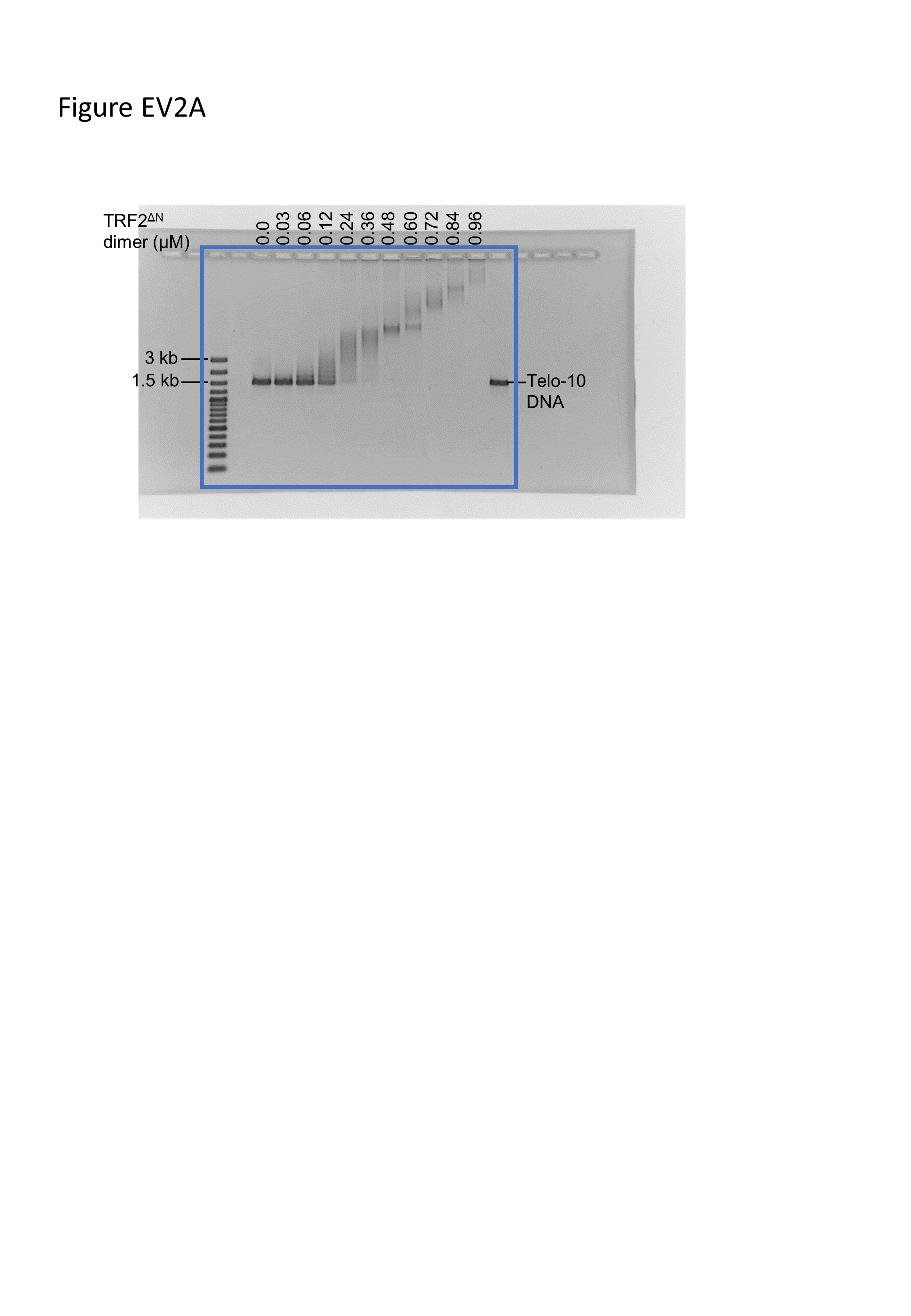

Supplement: Supplementary file 8 — Expanded View Figures Source Data [file 44318_2023_2_MOESM8_ESM.zip › EMBOJ-2023-114491_Source data_Expanded View/Figure Expanded View 2/EV2A/Image data_Figure EV2A.tif]

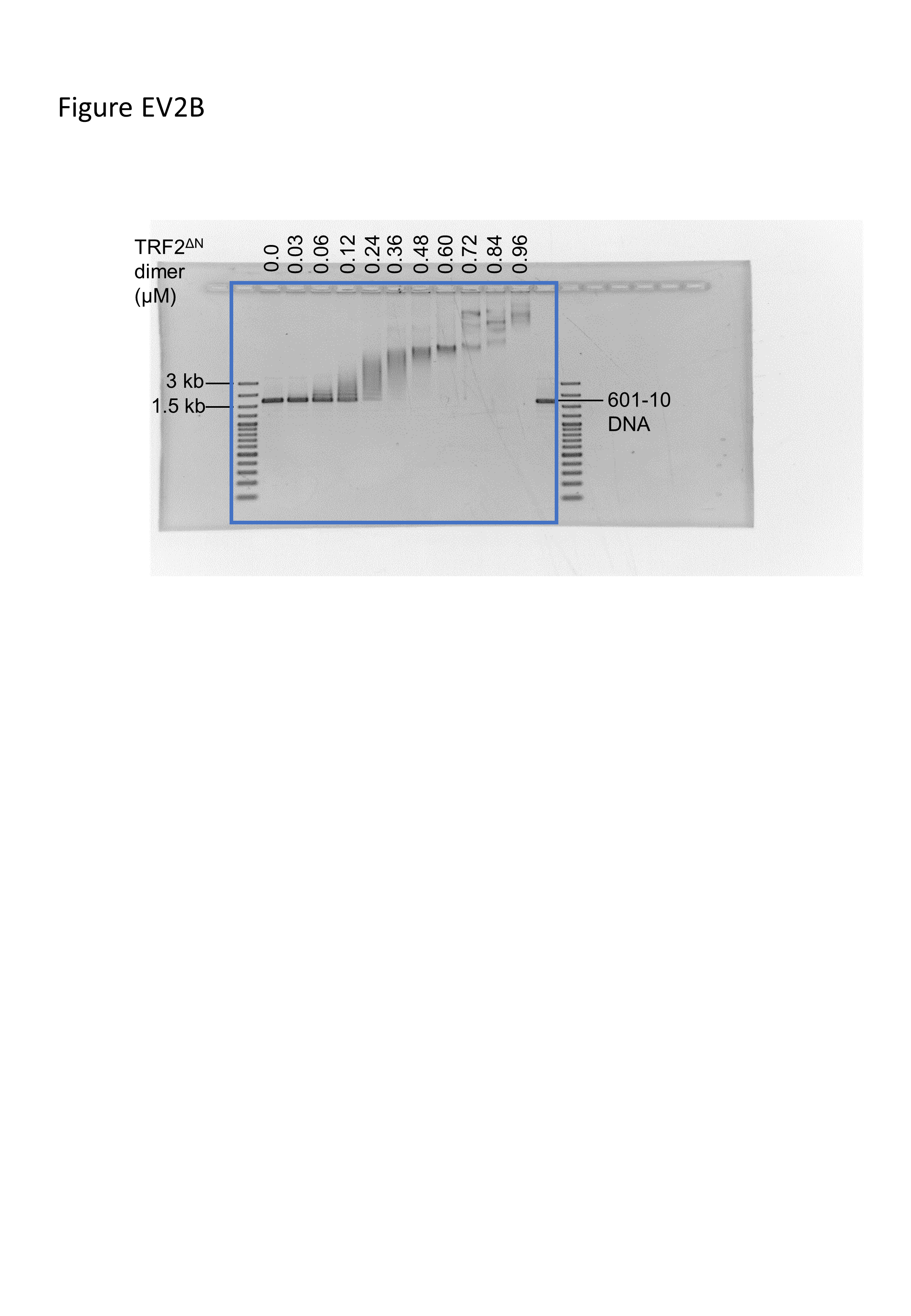

Supplement: Supplementary file 8 — Expanded View Figures Source Data [file 44318_2023_2_MOESM8_ESM.zip › EMBOJ-2023-114491_Source data_Expanded View/Figure Expanded View 2/EV2B/Image data_Figure EV2B.tif]

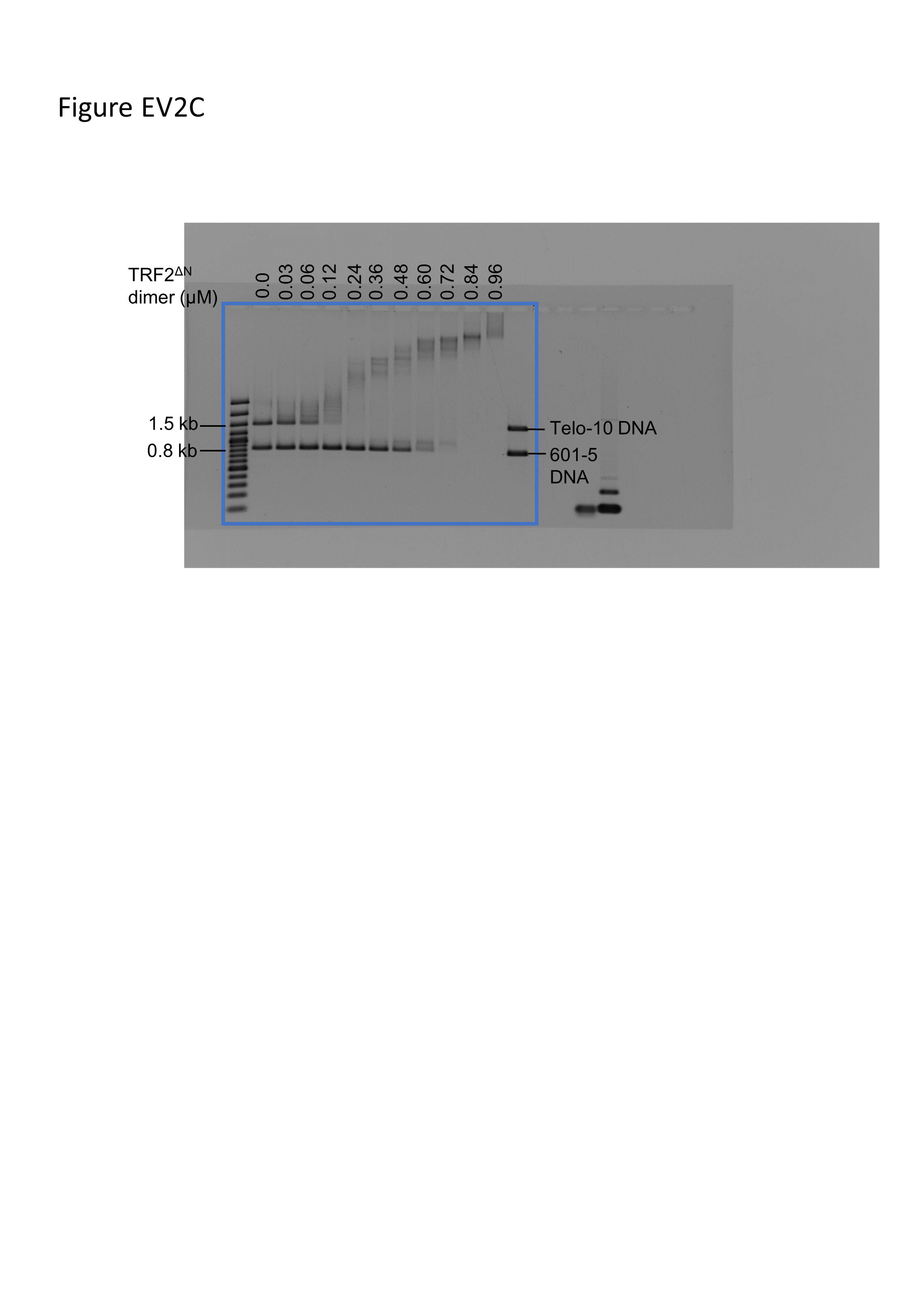

Supplement: Supplementary file 8 — Expanded View Figures Source Data [file 44318_2023_2_MOESM8_ESM.zip › EMBOJ-2023-114491_Source data_Expanded View/Figure Expanded View 2/EV2C/Image data_Figure EV2C.tif]

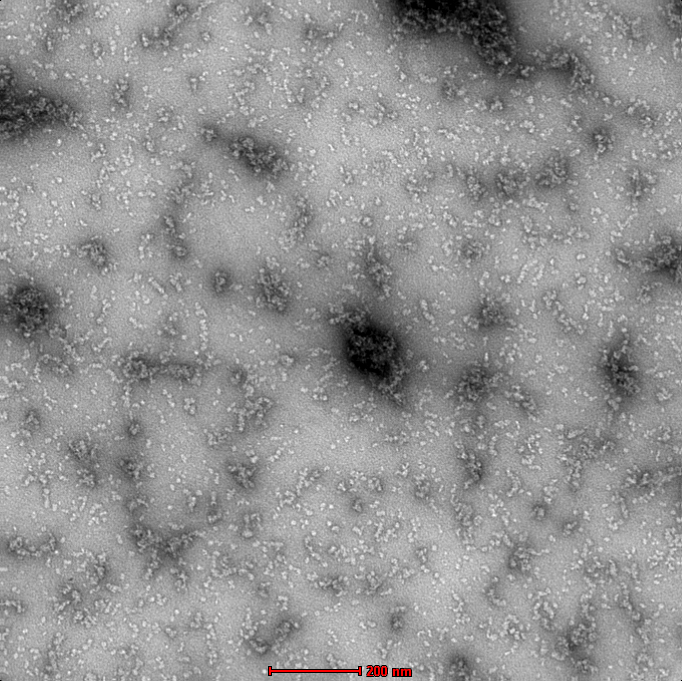

Supplement: Supplementary file 8 — Expanded View Figures Source Data [file 44318_2023_2_MOESM8_ESM.zip › EMBOJ-2023-114491_Source data_Expanded View/Figure Expanded View 3/EV3D/Imagedata_FigEV3D_Telo-10_0p1_TRF2.bmp]

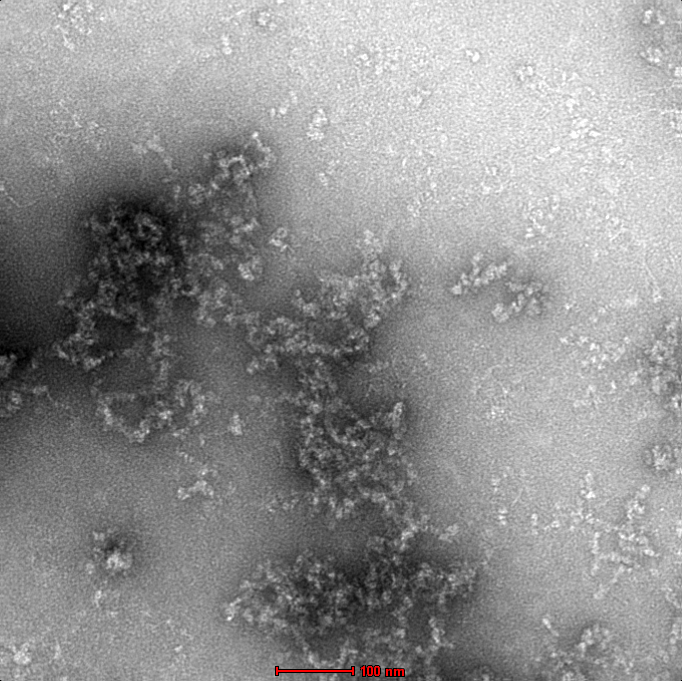

Supplement: Supplementary file 8 — Expanded View Figures Source Data [file 44318_2023_2_MOESM8_ESM.zip › EMBOJ-2023-114491_Source data_Expanded View/Figure Expanded View 3/EV3E/Imagedata_FigEV3D_Telo-10_0p4_TRF2.bmp]

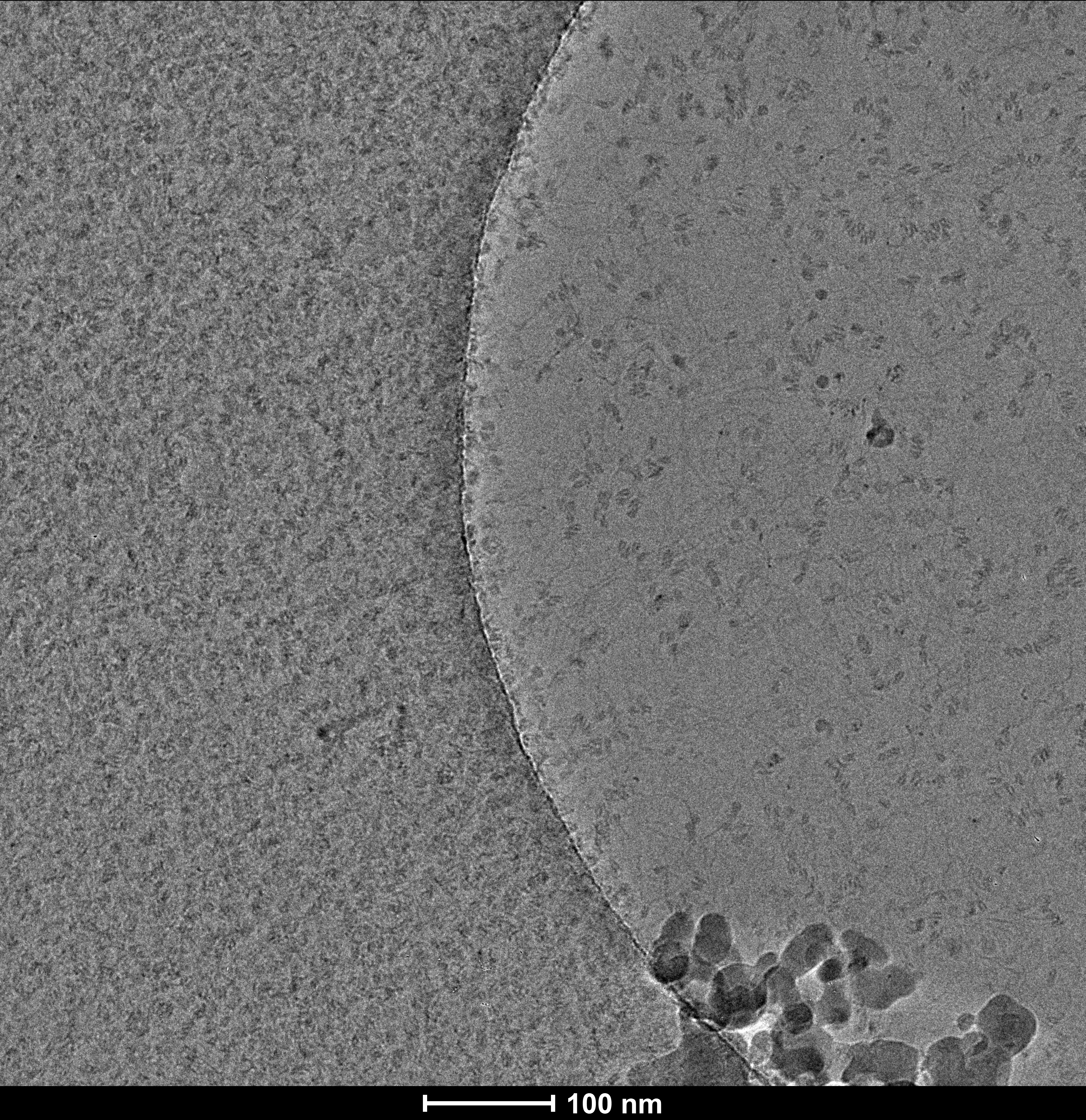

Supplement: Supplementary file 8 — Expanded View Figures Source Data [file 44318_2023_2_MOESM8_ESM.zip › EMBOJ-2023-114491_Source data_Expanded View/Figure Expanded View 3/EV3F/Imagedata_FigEV3D_Telo-4_0p5_TRF2.bmp]
