# Supplementary material for: The shelterin component TRF2 mediates columnar stacking of human telomeric chromatin
Source: EMBO J. 2023 Dec 14;43(1):87–111. doi: 10.1038/s44318-023-00002-3 (PMC10883271; doi:10.1038/s44318-023-00002-3)
Supplement: Supplementary file 9 — Expanded View Figures [file 44318_2023_2_MOESM9_ESM.pdf]

Expanded View Figures

Figure EV1. TRF2$^{\Delta N}$ binds telomeric and 601 nucleosome arrays under different salt conditions.

(**A**, **B**) EMSA analyses TRF2$^{\Delta N}$ binding to (**A**) Telo-10 and (**B**) 601-10 array in a solution containing 20 mM HEPES pH 7.4, 5 mM NaCl, 0.075 mM EDTA, and 0.75 mM DTT. TRF2$^{\Delta N}$ binding was done with a Telo-10 array at HO/DNA ratio of 1.05 or 601-10 array at HO/DNA ratio of 1.1 in the presence of 4% Cr_{147} competitor DNA. The data shown are representatives of three technically replicated experiments. (**C**, **D**) EMSA analyses TRF2$^{\Delta N}$ binding to (**C**) Telo-10 and (**D**) 601-20 array in a solution containing 20 mM HEPES (pH 7.4), 62.5 mM NaCl, 0.75 mM DTT and 0.075 mM EDTA. TRF2$^{\Delta N}$ binding was done with a Telo-10 array at HO/DNA ratio of 1.05 or 601-20 DNA with 4% Cr_{147} competitor DNA. The data shown are representatives of three technically replicated experiments. (**E**, **F**) EMSA analyses TRF2$^{\Delta N}$ binding to Telo-10 (**E**) or 601-20 (**F**) array in near-physiological conditions containing 20 mM HEPES (pH 7.4), 100 mM NaCl, 50 mM KCl, 1 mM $MgCl_2$, 0.1 mM EDTA, 1 mM DTT, 0.5 mg/ml BSA, 5% (v/v) glycerol and 0.1% (v/v) NP-40. TRF2$^{\Delta N}$ binding was done with a Telo-10 array at HO/DNA ratio of 1.05 or 601-20 DNA with 4% Cr_{147} competitor DNA. The data shown are representatives of three technically replicated experiments.

© The Author(s)

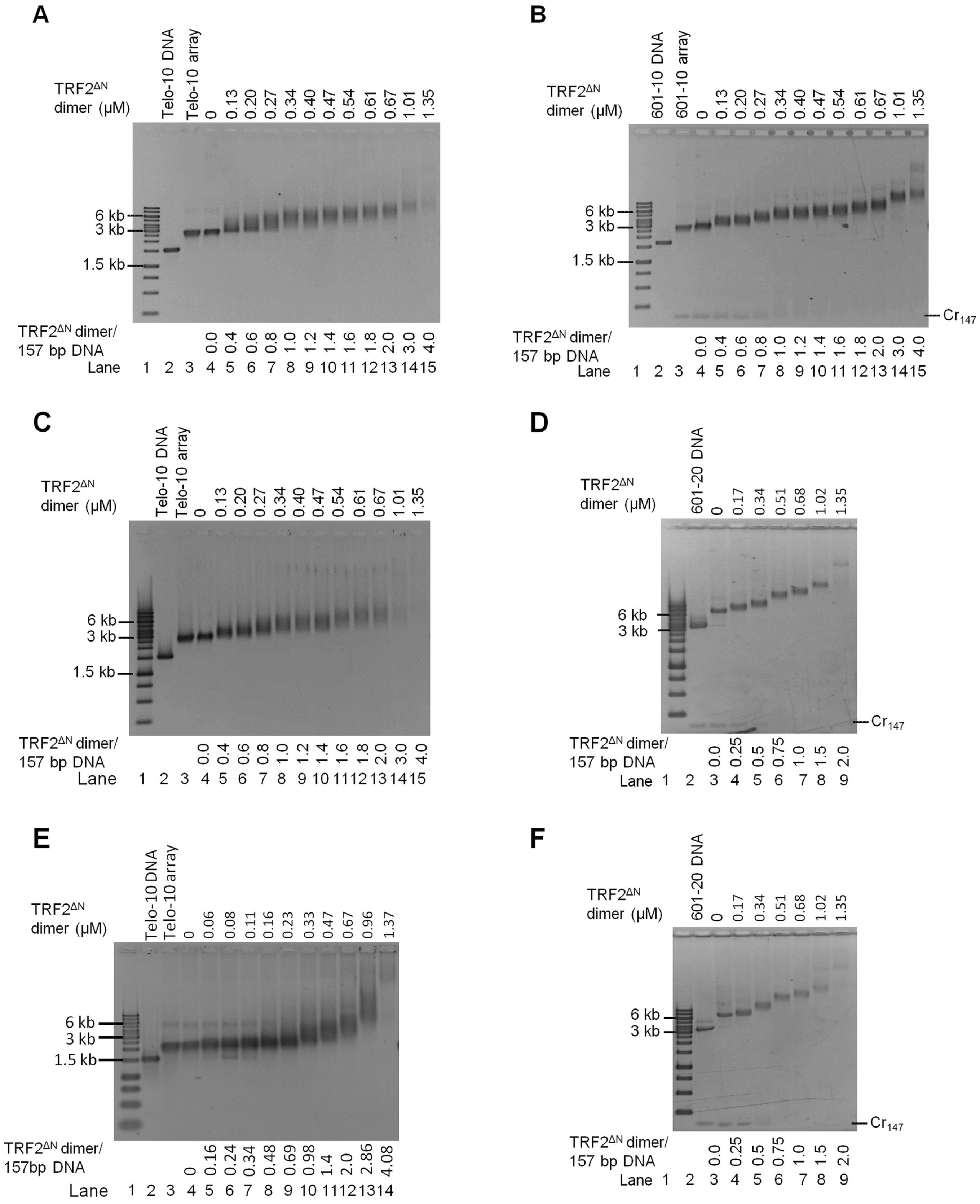
A
Telo-10 DNA
Telo-10 array
TRF2$^{\Delta N}$ dimer (μM)
0 0.13 0.20 0.27 0.34 0.40 0.47 0.54 0.61 0.67 1.01 1.35
6 kb
3 kb
1.5 kb
TRF2$^{\Delta N}$ dimer/ 157 bp DNA
0.0 0.4 0.6 0.8 1.0 1.2 1.4 1.6 1.8 2.0 3.0 4.0
Lane 1 2 3 4 5 6 7 8 9 10 11 12 13 14 15
B
601-10 DNA
601-10 array
TRF2$^{\Delta N}$ dimer (μM)
0 0.13 0.20 0.27 0.34 0.40 0.47 0.54 0.61 0.67 1.01 1.35
6 kb
3 kb
1.5 kb
Cr_{147}
TRF2$^{\Delta N}$ dimer/ 157 bp DNA
0.0 0.4 0.6 0.8 1.0 1.2 1.4 1.6 1.8 2.0 3.0 4.0
Lane 1 2 3 4 5 6 7 8 9 10 11 12 13 14 15
C
Telo-10 DNA
Telo-10 array
TRF2$^{\Delta N}$ dimer (μM)
0 0.13 0.20 0.27 0.34 0.40 0.47 0.54 0.61 0.67 1.01 1.35
6 kb
3 kb
1.5 kb
TRF2$^{\Delta N}$ dimer/ 157 bp DNA
0.0 0.4 0.6 0.8 1.0 1.2 1.4 1.6 1.8 2.0 3.0 4.0
Lane 1 2 3 4 5 6 7 8 9 10 11 12 13 14 15
D
601-20 DNA
TRF2$^{\Delta N}$ dimer (μM)
0 0.17 0.34 0.51 0.68 1.02 1.35
6 kb
3 kb
Cr_{147}
TRF2$^{\Delta N}$ dimer/ 157 bp DNA
0.0 0.25 0.5 0.75 1.0 1.5 2.0
Lane 1 2 3 4 5 6 7 8 9
E
Telo-10 DNA
Telo-10 array
TRF2$^{\Delta N}$ dimer (μM)
0 0.06 0.08 0.11 0.16 0.23 0.33 0.47 0.67 0.96 1.37
6 kb
3 kb
1.5 kb
TRF2$^{\Delta N}$ dimer/ 157bp DNA
0 0.16 0.24 0.34 0.48 0.69 0.98 1.4 2.0 2.86 4.08
Lane 1 2 3 4 5 6 7 8 9 10 11 12 13 14
F
601-20 DNA
TRF2$^{\Delta N}$ dimer (μM)
0 0.17 0.34 0.51 0.68 1.02 1.35
6 kb
3 kb
Cr_{147}
TRF2$^{\Delta N}$ dimer/ 157 bp DNA
0.0 0.25 0.5 0.75 1.0 1.5 2.0
Lane 1 2 3 4 5 6 7 8 9

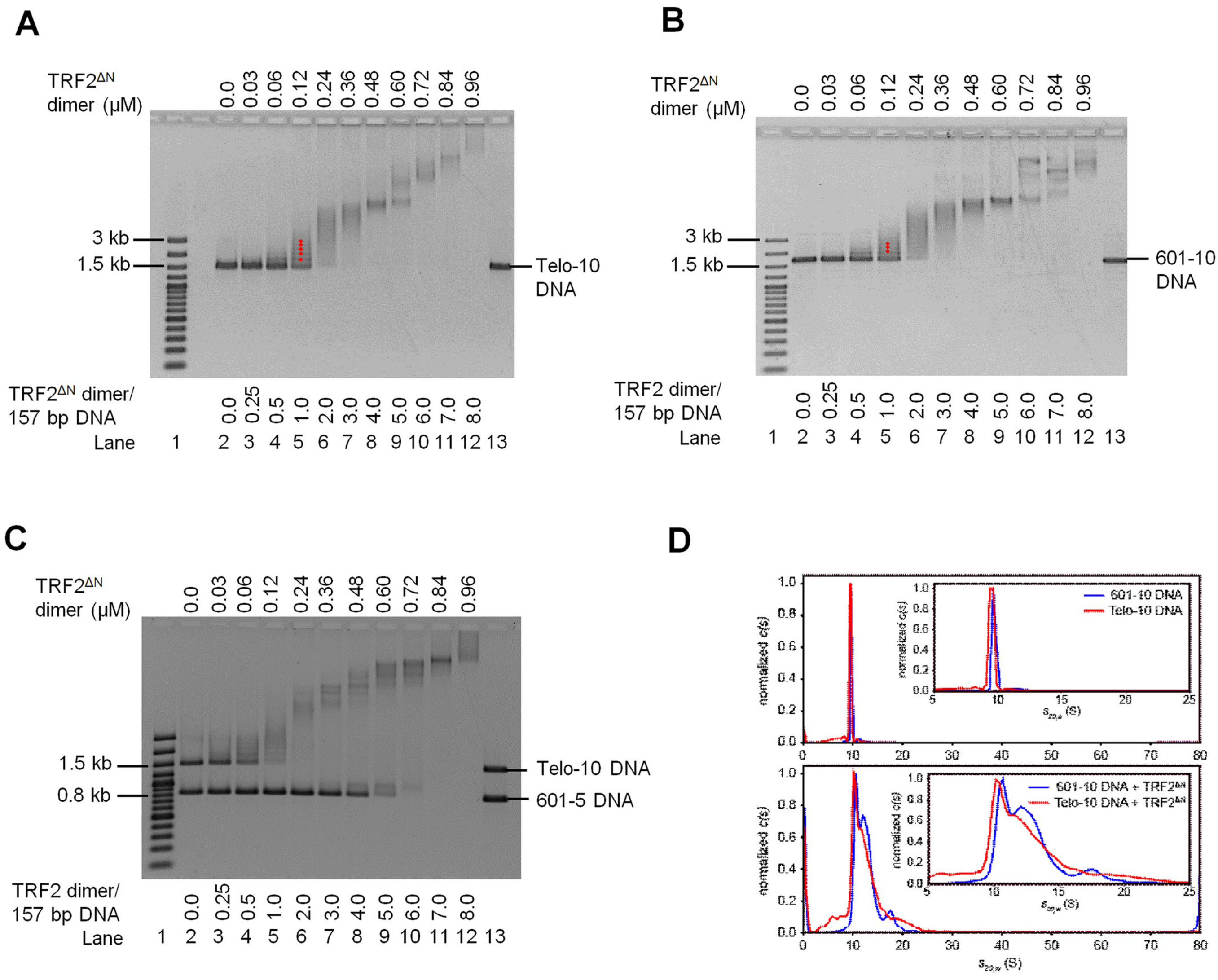

Figure EV2. TRF2$^{\Delta N}$ binds selectively to the telomeric sequence under competing conditions.

(**A**, **B**) EMSA analyses TRF2 binding to (**A**) Telo-10 DNA and (**B**) 601-10 DNA. The data shown are representatives of three technically replicated experiments. (**C**) EMSA analyses the DNA competition binding assay with TRF2 $^{\Delta N}$. The amount of Telo-10 and 601-5 DNA used in the competition binding assays were at a 1:1 mass ratio. The data shown are representatives of three technically replicated experiments. (**D**) Sedimentation coefficient (c(s)) distribution curves, obtained from AUC-SV data, for 601-10 and Telo-10 DNA (top) and 601-10 and Telo-10 DNA in the presence of TRF2$^{\Delta N}$ (bottom). Top panel: The *s*-value of the Telo-10 DNA in the absence of TRF2$^{\Delta N}$ dimers was 9.49 ± 0.02 S (red, top), which shifted to 10.4 ± 0.2 S in the presence of 0.2 μM TRF2$^{\Delta N}$ dimers (corresponding to 0.6 TRF2$^{\Delta N}$ dimer/157 bp of DNA) (red, bottom). The *s*-value of the 601-10 DNA was 9.70 ± 0.02 S (blue, top), which shifted to 10.60 ± 0.03 S in the presence of 0.2 μM TRF2$^{\Delta N}$). Curves are the average of the three technically replicated experiments; *s*-values are means ± s.d.

 © The Author(s)

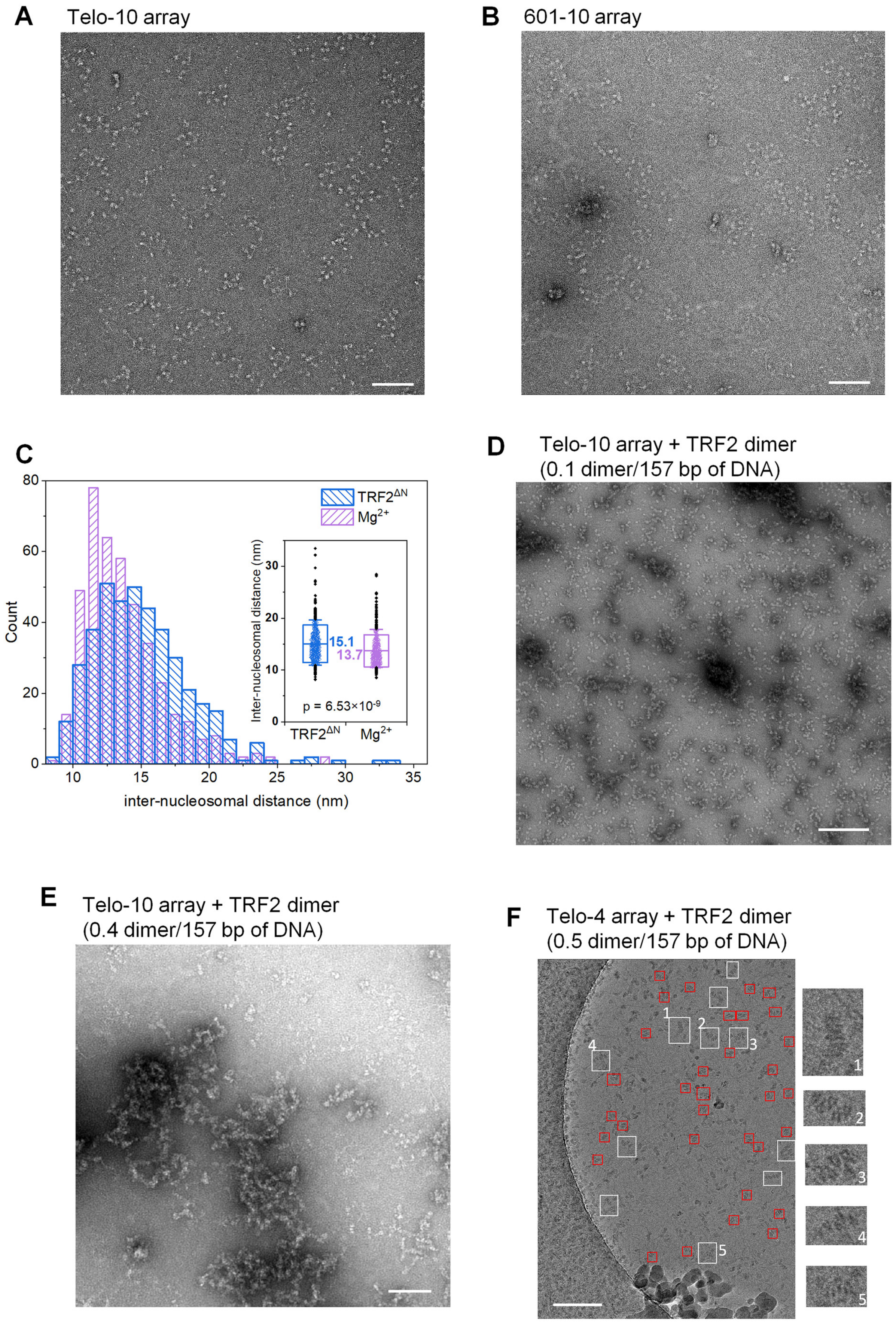
A
Telo-10 array
B
601-10 array
C
80
60
40
20
0
Count
10
15
20
25
30
35
inter-nucleosomal distance (nm)
TRF2ΔN
Mg2+
Inter-nucleosomal distance (nm)
30
20
10
0
15.1
13.7
p = 6.53×10-9
TRF2ΔN
Mg2+
D
Telo-10 array + TRF2 dimer
(0.1 dimer/157 bp of DNA)
E
Telo-10 array + TRF2 dimer
(0.4 dimer/157 bp of DNA)
F
Telo-4 array + TRF2 dimer
(0.5 dimer/157 bp of DNA)
1
2
3
4
5

Figure EV3. TRF2$^{\Delta N}$-telomeric array complex forms a homogeneous columnar arrangement.

Related to Figs. 2 and 3. (**A**) Representative negative-stained EM micrographs of Telo-10 array in the absence of TRF2$^{\Delta N}$ dimer. Scale bar: 100 nm. The data shown are representatives of three technically replicated experiments. (**B**) Representative negative-stained EM micrographs of 601-10 array without TRF2$^{\Delta N}$ dimer. Scale bar: 100 nm. The data shown are representatives of three technically replicated experiments. (**C**) Histogram plot for the inter-nucleosomal distance measured from 601-10 array in the presence of TRF2$^{\Delta N}$ (blue) and Mg^{2+} (purple). The insert shows the box plots overlaid with the data points (diamond symbols); statistical analysis was performed using a two-sample *t* test with an equal variance; p value $= 6.53\times10^{-9}$. Mean values are indicated in the insert; boxes show mean ± s.d.; whiskers indicate the 10-90% data range; black diamond symbols are outliers' data points. For 601-10 array in the presence of TRF2$^{\Delta N}$: $n = 413$, mean = 15.1 nm, s.d. = 3.6 nm and range = 8.2–33.4 nm. For 601-10 array in the presence of Mg^{2+}: $n = 419$, mean = 13.7 nm, s.d. = 3.1 nm and range = 8.5–28.4 nm. The data presented are from three technically replicated experiments. (**D**) Representative negative-stained EM micrographs of Telo-10 array in the presence of full-length TRF2 dimer at the ratio of 0.1 dimer/157 bp of telomeric DNA showing induction of columnar conformation. Scale bar: 200 nm. The data shown are representatives of three technically replicated experiments. (**E**) Representative negative-stained micrographs of Telo-10 array in the presence of full-length TRF2 dimer at the ratio of 0.4 dimer/157 bp of telomeric DNA equivalent to Fig. 2A demonstrating columnar conformation. Scale bar: 100 nm. The data shown are representatives of three technically replicated experiments. (**F**) Representative cryo-EM micrographs of Telo-4 in the presence of 0.5 TRF2 dimer/157 bp of DNA, white boxes indicate compact columnar fibres with three or more stacked nucleosomes, and red boxes indicate selected dimer stacks. Blow-ups 1–5 show columnar Telo-4 fibres in the presence of TRF2. Scale bar: 100 nm. The data shown are representatives of three technically replicated experiments.

 © The Author(s)

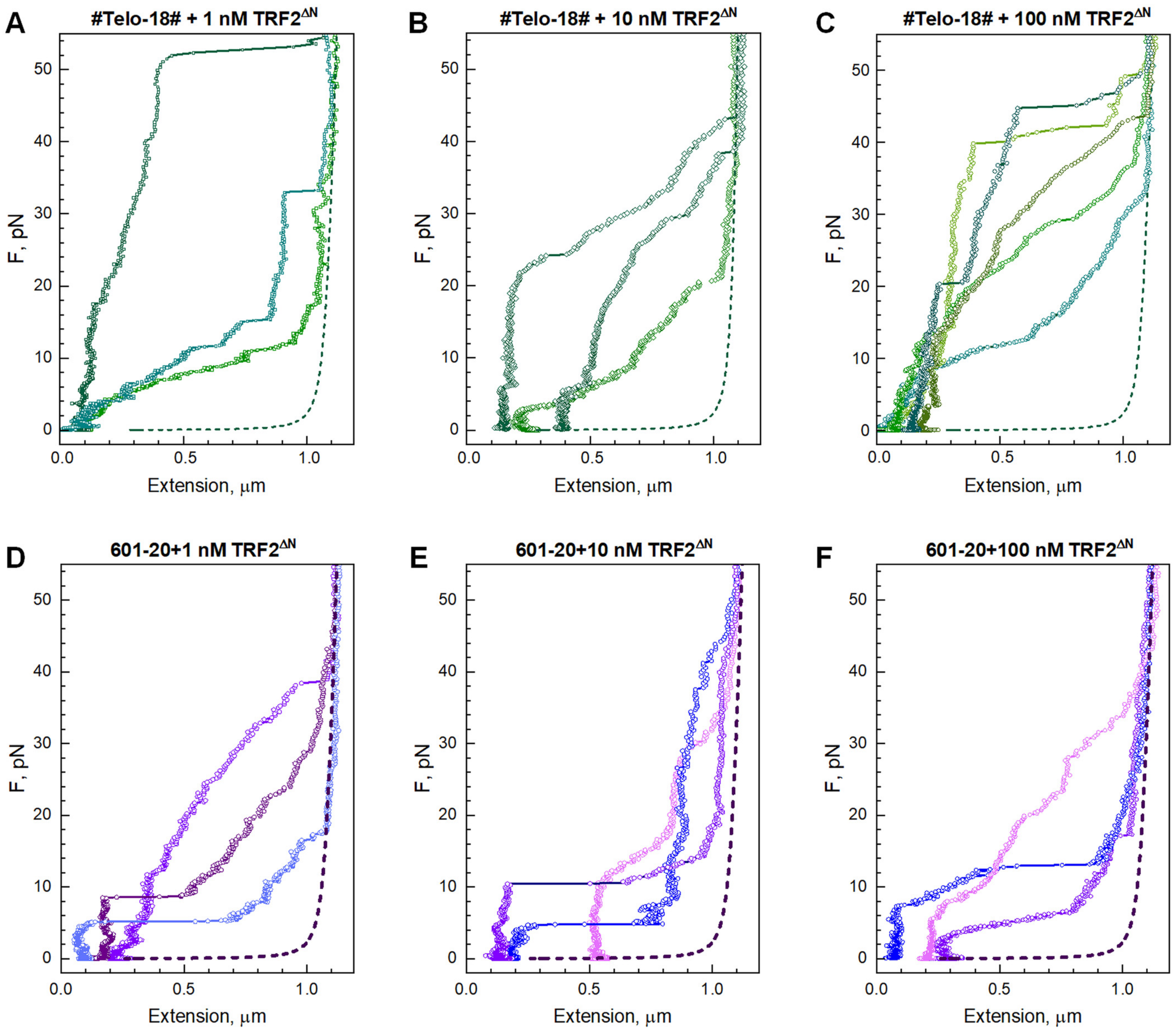

Figure EV4. Examples of stretching curves of the #Telo-18# and 601-20 arrays which cannot be reliably fitted by the current statistical mechanics model.

Related to Figs. 4 and 5. (**A–C**) Stretching curves of the #Telo-18# in the presence of 1 nM (**A**), 10 nM (**B**), and 100 nM (**C**) of the TRF2$^{\Delta N}$ dimer added to the flow cell. Dashed lines show stretching of the 3242 bp bare DNA calculated by the WLC model. (**D–F**) Stretching curves of the 601-20 in the presence of 1 nM (**D**), 10 nM (**E**), and 100 nM (**F**) of the TRF2$^{\Delta N}$ dimer added to the flow cell. Dashed lines show stretching of the 3242 bp bare DNA calculated by the WLC model.

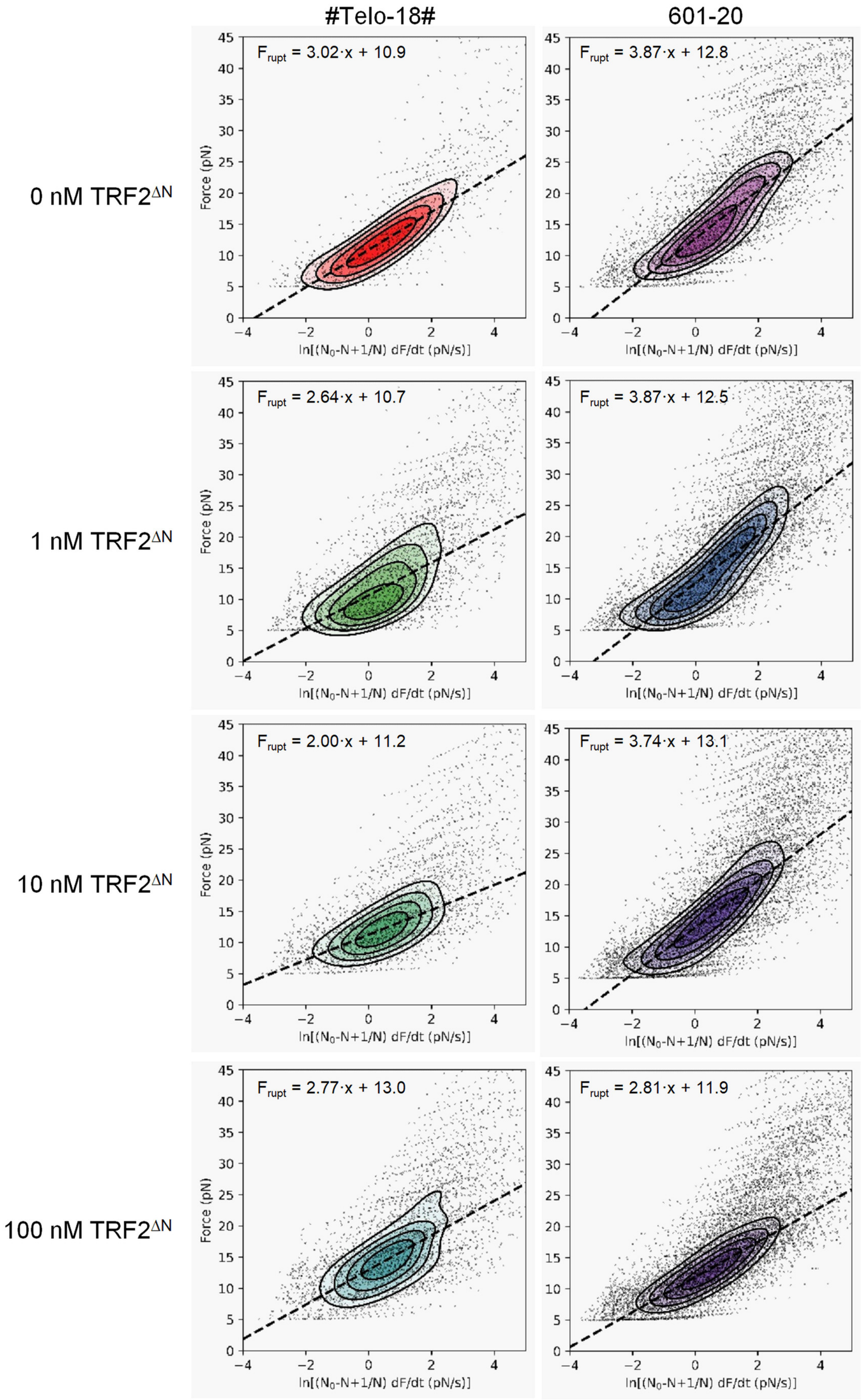
#Telo-18#
601-20
0 nM TRF2ΔN
1 nM TRF2ΔN
10 nM TRF2ΔN
100 nM TRF2ΔN
F rupt = 3.02·x + 10.9
F rupt = 3.87·x + 12.8
F rupt = 2.64·x + 10.7
F rupt = 3.87·x + 12.5
F rupt = 2.00·x + 11.2
F rupt = 3.74·x + 13.1
F rupt = 2.77·x + 13.0
F rupt = 2.81·x + 11.9
Force (pN)
ln[(N0-N+1/N) dF/dt (pN/s)]

 © The Author(s)

Figure EV5. Dependence of rupture force events, F_{rupt}, on the rate of applied force and the number of nucleosomes in the #Telo-18# and 601-20 arrays.

Related to Fig. 5F. According to the equation (Pope et al, 2005): $F_{rupt} = \frac{k_B T}{d}\left\{\ln\left[\left(N_0 - N - 1/N\right)\frac{dF}{dt}\right] - \ln\left(\frac{k_B T k_{off}}{d}\right)\right\}$ the rupture force (F_{rupt}) events depend on the rate of applied force (dF/dt), the number of nucleosomes at the initial moment (N_0) and the moment of rupture (N). In the equation above, d is the distance between the bound state and the activation barrier peak along the direction of the applied force; k_{off} is the rate constant for nucleosome rupture under zero external force; k_B is the Boltzmann constant, T is temperature. Note, that the pre-factor was added in the logarithm, as compared to (Pope et al, 2005), to adjust for force clamp mode as opposed to optical tweezers that operate in position clamp mode. Values of the d and $k_{off,}$ which are displayed in Fig. 5F of the main text, were determined from the slope and intercept of the linear fit in coordinates F_{rupt} versus $x = \ln\left[\left(N_0 - N - 1/N\right)\frac{dF}{dt}\right]$. In each graph, points indicate rupture events, and coloured contours highlight densities of the rupture distribution (2D histograms). For a more representative linear fit, the large impact of outliers was reduced by including only datapoints in bins that exceed a threshold of 0.5 times the peak value in the 2D histogram. The coloured contours indicate the areas of points included in the linear fitting; this 0.5-maximum cut-off discards about 50% of the datapoints. Lines show the linear fit of the data. The left-hand column of four graphs presents the data collected for the #Telo-18# arrays as indicated at the top; four graphs on the right are the results for the 601-20 arrays. Concentrations of the TRF2$^{\Delta N}$ dimer in the flow cell are 0, 1, 10, and 100 nM, as indicated in the four rows of two graphs. Numerical values (including mean ± s.d. and numbers of measured points, n) are given in Appendix Table S2.
